# Ethics based educational interventions on end-of-life care for undergraduate nursing students: A scoping review

**DOI:** 10.1016/j.ijnsa.2025.100465

**Published:** 2025-12-03

**Authors:** Saeko Kutsunugi, Satoko Ono, Misae Ito, Kaho Suda, Siu Ling Chan, John Tai Chun Fung, Claudia Kam Yuk Lai, Kyoko Murakami

**Affiliations:** a Yamaguchi University Graduate School of Medicine, Yamaguchi, Japan; b Nippon Medical School, Tokyo, Japan; c The University of Hong Kong, Pokfulam, Hong kong; d The Hong Kong Polytechnic University, Hung Hom, Hong Kong

**Keywords:** Baccalaureate education, End-of-life care, Nursing ethics, Teaching methods, Terminal care, Review, Simulation

## Abstract

**Background:**

Nurses face various ethical dilemmas and conflicts in end-of-life care; however, there is no evidence of effective undergraduate ethics education on the topic settings to address them.

**Objectives:**

This review aimed to describe educational interventions for end-of-life care focused on ethical content in undergraduate nursing education and their outcomes for students.

**Design:**

This review used Arksey and O'Malley’s five-stage scoping process.

**Methods:**

This study used the Ovid (CINAHL and MEDLINE) and PubMed bibliographic databases from 2012 to 2023. We included literature on qualitative, quantitative, and mixed-methods research that could provide information about ethical end-of-life care (EOLC) education interventions in undergraduate nursing programs. We excluded review articles to avoid duplication. The search string included ("education, nursing" OR "education, nursing, baccalaureate") AND ("ethics") OR ("terminal care" OR "hospice care" OR "end of life care" OR "palliative care"). The scope of this study was limited to studies on educational interventions for EOLC ethics in undergraduate nursing education.

**Results:**

The search yielded 4190 articles, of which 25 were included in the analysis. Fourteen of the 25 studies were conducted in the USA, whereas the others were conducted in various parts of the world. No intervention research articles were found that focused on EOLC ethics itself, which was either incorporated as part of the nursing education for EOLC or included in ethics education as an EOLC case study. Of these, 17 studies included lectures to teach EOLC ethics, and four used scenarios and case studies as teaching methods. Twelve studies used scenarios for simulation education. Seven studies combined lectures and simulations. The lectures improved students' knowledge and attitudes toward death, and their confidence in EOLC. As a result of the simulation intervention, in addition to improvements in knowledge and attitudes, participants gained communication skills, comfort with caregiving, confidence, self-efficacy, and the ability to reflect on their attitudes.

**Conclusions:**

The results suggest that while lectures are effective for acquiring ethical knowledge and attitudes related to EOLC based on ethical competency, simulations are more effective for acquiring practical skills such as communication, self-efficacy, and reflection. Incorporating ethical issues in simulations is hoped to broadly develop ethical practice competencies.

**What is already known**

● Undergraduate nursing students are inadequately prepared for end-of-life care.

● Nursing students face various ethical dilemmas and conflicts in end-of-life care.

● There is no clear teaching methodology for ethics education in end-of-life care.

Alt-text: Unlabelled box

**What this paper adds**

● No studies focused specifically on ethics education in end-of-life care.

● It highlights an important knowledge gap in baccalaureate nursing education.

● Nurse educators should consider effective approaches in nursing education

● Facilitate students to develop ethical competence and decision-making abilities

Alt-text: Unlabelled box

## 1 Introduction

When a patient is facing the end of life, nurses close to the patient are in an important position to improve their quality of life. Undergraduate nursing students are also motivated to support patients and their families and create a peaceful environment in which they can experience the end of life together (Abelsson and Willman, 2020; Ranse et al., 2016).

With an aging population and an increasing number of people living with chronic diseases, and traditional palliative care focused mainly on pain and symptom management for cancer patients, terminal care for end-of-life care (EOLC) is inadequate. The World Health Organization (WHO, 2023) defines palliative care as “an approach that improves the quality of life of patients – adults and children – and their families who are facing problems associated with life-threatening illness. It prevents and relieves suffering through the early identification, impeccable assessment, and treatment of pain and other problems, whether physical, psychosocial, or spiritual.” While this primarily focuses on medical aspects, a more comprehensive and culturally sensitive perspective that considers physical and psychosocial support and the existential and relational aspects of care is also necessary. The concept of “End-of-Life Care” is becoming increasingly important in considering the “good death” that each individual will eventually face and in providing high-quality care (Izumi et al., 2012). Although it is difficult to predict prognosis in the final stages of a person's life, there is a need for care that considers the course of the disease, such as sudden death, terminal illness, multiple organ failure, and frailty, each of which has a different duration and degree of deterioration (Lunney et al., 2002). EOLC is complex because it involves patients' physical, social, psychological, and spiritual needs. Caring for patients at the end of life is a challenging task, both emotionally and physically. Furthermore, nurses face many ethical issues in end-of-life care (Esfandiari et al. 2023). Undergraduate nursing students and newly qualified nurses reported that their education did not adequately prepare them to deal with the complexities of patient deaths (Kilcullen and Ireland, 2017; Murnane et al., 2023). Sensitivity to ethical issues and judgment skills are most effectively acquired and maintained through education and training, and therefore must be closely integrated into nursing curricula (Kim and Park, 2019). In the current clinical settings, undergraduate nursing students need to support end-of-life care for patients immediately after graduation, and there is a need to enhance ethical end-of-life care training in their nursing education.

## 2 Background

Death is one of the most vulnerable moments in life, and care at this time has been shown to improve quality of life more than that in usual nursing practice (Holmenlund et al., 2017). EOLC has become an important topic in the education and training of healthcare professionals (Donne et al., 2019).

Healthcare professionals, including nursing students, face ethical dilemmas or conflicts in EOLC because of the unpredictable nature of the patient’s physical and emotional condition, the social conditions of the family and community, the values of the patient and family, and even the values of themselves providing care. Examples of ethical dilemmas include decisions regarding resuscitation, artificial ventilation, artificial nutrition and hydration, terminal sedation, withholding and withdrawing treatment, euthanasia, and physician-assisted suicide (Akdeniz et al., 2021). The ethical challenges faced by healthcare professionals daily include applying ethical principles, providing clinical care, working with families, being involved in organizational structures, meeting societal values and expectations, and the philosophy of palliative care (Schofield et al., 2021). When newly graduated nurses enter the clinical environment, they must be prepared to provide quality care while navigating complex relationships with patients, their families, physicians, nurses, and other healthcare team members. Therefore, nursing students are expected to be well-trained in the ethical components of end-of-life care education.

Nurse educators recognize the importance of fostering ethical attitudes among students (Zolkefli et al., 2024). Ethical sensitivity—recognizing situations that make people vulnerable and insight into the ethical consequences of one's practice—is a prerequisite for addressing ethical dilemmas in complex healthcare contexts (Kim et al., 2004, 2016). Dealing with these ethical issues requires nurses to possess ethical competencies related to sensitivity, knowledge, reflection, decision-making, action, and behavior (Lechasseur et al., 2018). In this context, structured educational programs integrating ethical reflection into clinical practice are essential. The End-of-Life Nursing Education Consortium (ELNEC) was established in 2000 by the American Association of Colleges of Nursing (AACN) and the City of Hope. It includes modules focused on ethical and legal issues in ELOC care and aims to cultivate nurses' ethical sensitivity and moral courage (Matzo et al., 2004). Currently utilized in over 100 countries and is being progressively incorporated into undergraduate curricula. While programs such as ELNEC highlight the potential of clinically grounded ethical education, further research is needed to clarify the specific implementation of ethical education for nursing students.

Despite advances in nursing science and growing social, research, and clinical ethical concerns, there is little common understanding of the role of ethics education in nursing education curricula or the outcomes that can be achieved through ethics education (Katelin Hoskins, 2018). Case studies and discussions are often used as approaches to ethics education in undergraduate nursing education (Thiel et al., 2013). A growing body of literature demonstrates the educational value of various simulation methods, such as standardized patient and low-fidelity simulations that are essential for improving nursing students' skills (Li and Li, 2024). Simulations are well-designed activities or case studies that mimic real or potential scenarios students may encounter (Lioce, 2020). Simulations can effectively improve learning outcomes, such as knowledge, attitudes (Lee and Lee. 2022), psychomotor, reactive, cognitive, and emotional skills (Kim et al., 2016) and are the safest solution for bridging the gap between nursing theory, knowledge, and practice regarding EOLC (Gillan et al., 2021; Nunes and Harder, 2019). However, the abovementioned simulation methods exist in various forms and numbers, with advantages and disadvantages noted for each (Alwawi and İnkaya, 2023). To our knowledge, there is no clear teaching methodology for ethics education for undergraduate nursing students of basic nursing education programs in complex and highly individualized EOLC. This review addressed two questions:

1. What educational interventions for nursing EOLC ethics are used in nursing undergraduate education?
2. What knowledge, attitudes, and ethical competencies do undergraduate nursing students acquire from their EOLC education?

### 2.1 Definition of terms

End-of-life care is "to assist persons facing imminent or distant death to have the best possible quality of life until the end of their lives, regardless of their medical diagnosis, health status, or age" (Izumi et al., 2012). As learners at the entry level of professional education, nursing students will likely encounter various end-of-life situations throughout their careers. Therefore, we have adopted this definition, which encompasses a longer time frame than the definition of palliative care and includes nursing care for people in diverse health conditions.

In this paper, we adapted the definition of “ethical competence” defined by Lechasseur et al. (2018) as “the ability to identify ethical issues, knowledge of the ethical and moral aspects of care, reflection on one's own knowledge and actions, and the ability to make wise decisions and manage ethically challenging work situations appropriately.”

### 2.2 Objectives

This scoping review aimed to describe educational interventions for EOLC focused on ethical content in undergraduate nursing education and their outcomes for students.

## 3 Methods

### 3.1 Design

The authors adopted Arksey and O'Malley’s five-stage scoping review process (Arksey and O'Malley, 2005). The Preferred Reporting Items for Systematic Reviews and Meta-Analyses (PRISMA) Extension for Scoping Reviews (Tricco et al., 2018) guided the reporting of this review. The methodological approach for this review included five steps: (1) identifying the research question, (2) identifying relevant studies, (3) selecting studies, (4) mapping data, and (5) collating and summarizing the findings. The review team comprised eight nursing academics and researchers. All the authors were involved in discussing and formulating the research questions.

### 3.2 Eligibility criteria

The eligibility criteria were based on best practice guidance from the Joanna Briggs Institute (JBI) Scoping Review Methodology Group (Peters et al., 2022), which included the participants, concept, and context of this review: (1) participants were baccalaureate nursing students; (2) concepts were educational approaches to nursing ethics for EOLC; and (3) the context was a baccalaureate nursing program (Table 1).

**Table 1:** Inclusion and exclusion criteria.

PICoS	Inclusion Criteria	Exclusion Criteria
Participants	Baccalaureate nursing students	Diploma nursing school students, faculty, registered nurses, charge nurses
Phenomenon of Interest	How nursing ethics at EOLC was being taught, and to what effect in the Baccalaureate nursing programme	Not end-of-life care educational intervention
Context	Baccalaureate nursing education	Settings others than BSN program
Types of studies	Qualitative, quantitative, and mixed-methods studies	Conference abstracts, thesis and dissertations, review papers, and editorials

The following criteria were applied: peer-reviewed articles, such as research reports (including qualitative, quantitative, and mixed-methods research) that clearly describe interventions in ethics education in EOLC for nursing students (to be consistent with the study objectives). Papers that clearly stated ‘ethics’ in their lecture content were selected as papers that provide ethics education. Furthermore, papers that focused on ethical issues in case studies such as simulations were also selected as papers that provide ethics education. The following papers were excluded: implementation, guidelines or standards, conceptual analyses, theoretical papers and policy documents. We limited our search to English-language papers published between 2012 and 2023 to consider the most recent data.

### 3.3 Information sources

The search strategy was developed by all authors. The initial phase of the search was conducted using CINAHL and Medline to identify keywords related to the review objectives. The term “End-of-Life Care” is not directly used in the WHO definition of palliative care. Nonetheless, its practices (acceptance of death, pain relief, family support, etc.) are comprehensively included. However, differences in perceptions among experts remain, and no consensus on terminology has been reached. The search terms included ("education, nursing" OR "education, nursing, baccalaureate") AND ("Ethics") OR ("terminal care" OR "hospice care" OR "end of life care "OR" palliative care"). For PubMed, 〈"education, nursing [MeSH Terms]" OR "education, nursing, baccalaureate [MeSH Terms]"〉 AND 〈"Ethics"〉 OR 〈“palliative care” OR "terminal care [MeSH Terms]” OR "hospice care [MeSH Terms]" OR "end of life care [Text Word]"〉 were used. The final search was conducted in August 2023.

### 3.4 Selection of sources of evidence

After removing duplicate citations, four authors (first to fourth authors) read the titles and abstracts of the studies identified through keyword searches and narrowed down the full-text review candidates. Subsequently, all eight authors were divided into two reviewer groups and read the full-text papers in a collaborative effort, selecting papers that aligned with the search strategy of this study. Both groups confirmed the inclusion of each study, and disagreements were resolved through discussion until a consensus was reached.

### 3.5 Charting the data and synthesis of results

All authors contributed to preparing the data tables. The data were tabulated, including names of the authors of the articles, year of publication, country/region, study aims and design, methodology, description of cases, study sample, outcomes, and summary of key findings (relevant to the review question). We also extracted details of implementation of intervention, if available. The extracted data were exported to an Excel file and reviewed by four authors (SK, SO, MI, and KS).

### 3.6 Study selection

**Fig 1:**
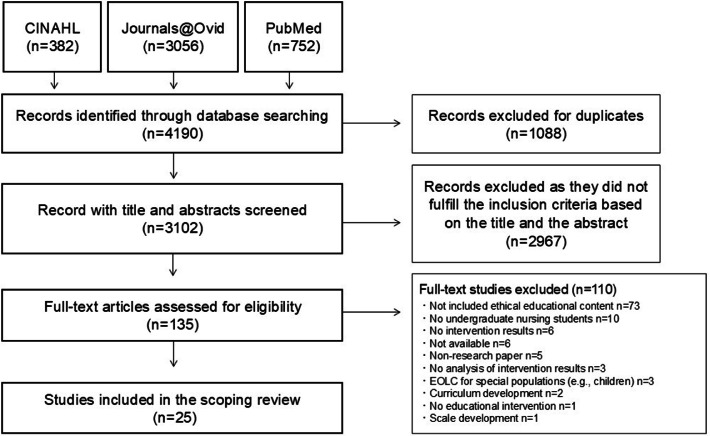
Preferred reporting items for systematic review and meta-analyses flowchart of literature search and inclusion.

Our search initially yielded 4190 studies; 1088 duplicates were removed. Then, the authors reviewed the abstracts of the remaining 3102 articles based on the inclusion and exclusion criteria. The remaining 135 articles were reviewed in full by all authors who worked in four groups (two per group); each author worked independently, and the partner checked their decisions. In cases of conflicting decisions, all the authors met and discussed different viewpoints to reach a consensus regarding the inclusion or exclusion of a particular article. This process excluded an additional 110 articles. The remaining 25 articles were eligible for inclusion in this review (Fig. 1). Most of the studies were conducted or reported by authors from the United States of America (USA) (*n* = 14), with the remainder from Spain (*n* = 2), the United Kingdom (UK) (*n* = 2), Sweden (*n* = 1), Norway (*n* = 1), Korea (*n* = 1), China (*n* = 1), South Africa (*n* = 1), Cameroon (*n* = 1), and Palestine (*n* = 1).

## 4 Results

In this section, we first describe the characteristics of ethics education identified in the papers then, we summarize the content identified in the papers for each section, including lectures, simulations, clinical training, other teaching methods and evaluation methods.

### 4.1 Characteristics of ethical education on end-of-life care

None of the articles focused on EOLC ethics education itself. EOLC ethics in nursing undergraduate education was either incorporated into EOLC education or taught end-of-life topics in ethics education. Table 2 presents the selected studies, program characteristics, educational methods, and key findings. The study designs were categorized as interventional pre-post studies (*n* = 10), two-arm pre-post comparative studies (*n* = 8), qualitative studies (*n* = 6), or cross-sectional mixed-method studies (*n* = 1) (Table 2). The number of participants ranged from 18 to over 400. The grade levels of the target students ranged from first-year students to seniors; some of the studies included RN-BSN students (Conner et al., 2014; Loerzel and Conner, 2016), while one included RNs and NPs (Orr et al., 2021).

**Table 2:** Characteristics of included studies.

Authors (year) Country	Study design	Aim	Participants	Educational Method						Key findings
				Method				Program characteristics	Evaluation method	
				Lecture	Simulation	Clinical practice	Other methods			
Alwawi et al. (2023) Palestine	RCT quasi-experimental study	To test the effects of a LFS in comparison to SPS in palliative care teaching	70 sophomore students	●	● LFS			Simulation after a regular 3-unit palliative care nursing course involving ethical issues. Comparison of SPS and a LFS. The simulation deals with communicating bad news (the progression of the disease) to the patient and his family.	PCQN; Learner Satisfaction and Self-confidence in Learning; Uniquely designed questionnaire items	Students' knowledge improved significantly on the post-test compared with the pre-test, without significant differences between both groups. The utilization of the two methods in students' clinical training for scenarios had the same effect on satisfaction and confidence. The skills of the SPS group improved significantly more than the LFS group. Both simulation modalities were effective for palliative care nursing students.
				●	● SPS					
Bailey et al. (2014) UK	Quasi-experimental study	To evaluate the impact of an educational workshop delivered as part of a critical care module prior to a six-week critical care clinical placement	21 junior students				Case study	One-day workshop using EOLC scenarios based on real-life situations in critical care that presented professional, ethical, and clinical challenges.	FATCOD; qualitative data of participants’ perceptions of the strengths and limitations of the program	Workshops that use case studies based on real-life episodes of EOLC can provide an effective learning opportunity that significantly improves the attitudes of nursing students to caring for the dying. Although many of them had felt unprepared and anxious about communicating with patients who were terminally ill, their relatives, and bereaved relatives, the post-test feedback reflected a positive attitude to the educational workshop. Raising awareness of the ethical, professional, and personal issues involved in EOLC and identifying practical steps for managing them enabled students to think about stage 2, "managing emotional labor," of the model.
Bassah et al. (2018) Cameroon	Pre-post qualitative descriptive study	To describe nursing students’ experiences and perceptions of a palliative care course	23 sophomore or junior students	●			Video Case study Interactive demonstration Small group discussion Role-play	The course consisted of 30 h of lectures (including ethical content), videos, case studies, interactive demonstrations, small group discussions, and role-play.	Analyze qualitative descriptions	The use of a variety of interactive educational strategies, including supervised clinical practice, was considered by nursing students as vital to enhancing learning in palliative care education.
Conner et al. (2014) USA	Two-arm pre-post test study	To evaluate the impact of an online death and dying course on attitudes and feelings	Two basic nursing program students, 118 RN-BSN program students, and three concurrent program students. No information on grades	●			Online resources YouTube videos Reading books Discussion Reflection	Comparison of a 16-week online course with an ELNEC-based Death and Dying course. The course, including ELNEC, consisted of reading assignments from the textbook, links to online resources, and YouTube videos related to the topic. The course content included ethical decision making and the video links were primarily used to present the pros and cons of ethical issues in EOL (e.g. physician assisted suicide, discontinuation of nutritional intake).	Analyze qualitative descriptions; FATCOD; DAP-R	An online death and dying course were effective in improving nursing students’ attitudes toward care of the dying and improving death acceptance for those without religious affiliation. Nurse researchers need to refine current understanding of EOLC and move the knowledge forward. Nurse educators need to scaffold EOL education to drive home the idea that it should be ongoing so nurses can become experts in EOLC.
				● Online course with no EOL content						
Loerzel et al. (2016) USA	Qualitative descriptive study	To reflect on this online elective course on death and dying, evaluate the knowledge gained through the course, and apply it to current and/or future practice	36 prelicensure and RN-BSN program students No information on grades	● Online course				Death and dying online course content included death and dying processes, ethical decision-making and legal aspects.	Analyze qualitative descriptions	Nursing students can become more confident in EOL care through online education.
Donnelly et al. (2017) USA	Two-arm pre-post test study	To determine if participation in an ethics consultation simulation increased knowledge of nursing ethics principles compared to students who were taught ethics principles in the traditional didactic format	145 junior or senior students		● 1-hour ethics consultation simulation			A comparison between a group who participated in a 1-hour ethics consultation simulation and a group who watched an approximately 60-minute video recording of an ethics consultation simulation. The content of the simulation concerned surrogate decision making for an elderly man who lacked decision making capacity and had a complex course of respiratory failure due to pneumonia.	Items from The Guide to the Code of Ethics for Nurses with Interpretive Statements written by Dr. Marsha Fowler	Nursing students’ knowledge of nursing ethics principles significantly improved from pre-test to post-test; however, there was no significant difference between the experimental and control groups' knowledge scores. An ethics consultation simulation demonstrated how ethical dilemmas are identified, nursing ethics principles are applied to the dilemmas, and appropriate recommendations are constructed.
							Shown a roughly 60-minute video recording of an ethics consultation simulation			
Escribano et al. (2021) Spain	Quasi-experimental study	To evaluate the effectiveness of a simulation programme using SPs in 12 complex scenarios linked to chronic and EOL situations	161 senior students	●	●			Eight sessions lasting 2.5 h each and HFS intervention with SPs. Case content included conflicts in family decision making.	Attitude Toward Communication Scale; SE-12; EHC-PS	Self-efficacy and communication skills significantly improved after completing the intervention, with no significant differences in attitude toward the communication variable. The initial scores on this scale were already high relative to the maximum possible score; therefore, there was not much room for improvement.
Glover et al. (2017) USA	Quasi-experimental study	To report the results to incorporate EOL and palliative care training using the ELNEC framework and concepts	53 senior students	●			Interactive learning activities	Clinical practice at the local hospice care center and interactive learning activities based on 2-day ELNEC courses. ELNEC modules contain ethical content.	ELNEC-KAT	ELNEC core course is an effective way to improve nursing students' knowledge of palliative and EOL care. Nurse educators may integrate content on palliative and EOL care into standard nursing curricula using the ELNEC core course
Goode et al. (2019) UK	Quantitative longitudinal study	To explore students' evaluation of EOLC learning within a three-year undergraduate adult nursing degree program	336 freshmen to junior students	●	●		Role-play Case-based learning	Curriculum includes content on ethical dilemmas and ethical decision making.	Analyze qualitative descriptions	The majority of students reported satisfaction with academic level of teaching and learning about EOL; students evaluated the usefulness of skills during EOL situations in both personal and professional lives. This illustrates how knowledge crosses over from the classroom to practice, and how personal development and values can be influenced by professional understanding.
Grossman (2013) USA	Quasi-experimental study	To develop an algorithm that would assist student and critical care nurses in facilitating a positive death for acutely ill dying patients.	50 senior students		●		Case study	Ethical decision making, including DNR, related to the presence or absence of advanced directives in cases.	ELNEC-KAT	The use of an algorithm that provided a consistent method for assessment during practice opportunities improved students' knowledge scores and their perceptions of comfort in caring for the dying acutely ill. The findings supported the need for nursing students to practice therapeutic communication regarding palliative care in various settings such as in a simulated critical care unit, interactive classroom with faculty-student discussions regarding dying critically ill patients and role-playing therapeutic communication skills in class with the assistance of an expert faculty member.
Hjelmfors et al. (2016) Sweden	Qualitative ethnographic study	To increase knowledge about EOL care simulation in nursing education by describing and evaluating the delivery of simulation	60 junior students		●			One-day complete simulation with three scenarios. The learning objectives of the EOL simulation were to be able to identify and consider spiritual and existential needs in relation to critical illness and dying, and to apply a professional approach to patients and families based on ethical reasoning.	Analyze qualitative descriptions	EOLC simulation provided nursing students with communication skills training in challenging situations and the opportunity to reflect on their own attitudes and feelings and discuss what factors can have an impact on such conversations.
Jablonski et al. (2020) USA	Qualitative descriptive study	To describe a simulation focusing on nursing process, clinical reasoning, communication skills, and therapeutic relationships	More than 400 senior students		●			EOL simulation after ELNEC program. ELNEC modules contain ethical content.	Analyze qualitative descriptions	Based on the reflections and feedback received from students after they experienced the death of a patient for the first time as a nurse, the simulation played a significant part in preparing them for EOLC. Themes included knowledge of the EOL experience, creating a calming environment, emotions felt, debriefing and reflection as catharses, and simulation design
Jo et al. (2015) Korea	Two-arm pre-post test study	To examine the effects of a humanistic EOL care course	39 senior students	●				Comparison of typical traditional lectures and humanistic EOLC courses. Humanistic EOLC courses include ethical issues.	Attitudes toward death, DAS; CAT	Attitudes toward death and communication skills appeared to increase in the experimental group. However, death anxiety did not significantly differ between the two groups. The humanistic EOLC course was effective in reducing negative attitudes toward death and increasing the communication skills of Korean nursing students.
				●			Build a model funeral home Group discussions Presentation Writing Role-play Share personal experiences			
Lewis-Pierre et al. (2019) USA	Two-arm pre-post test study & mixed-method study	To examine differences in attitudes toward care of the dying in two groups of students	136 freshmen	● Online module	●			Comparison of group with online module only vs. group with online module and simulation. A two-part hybrid simulation on the care of patients diagnosed with end-stage non-Hodgkin's lymphoma, with content on DNR.	FATCOD-B and original open-ended qualitative questions	Both the online module and simulation experiences demonstrated positive impacts of students' attitudes toward caring for the dying, but the addition of simulation appreared to provide a more hoistic and meaningful experience for the students where they could begin to build their personal understanding of death and dying and care for this population and their families through a constructive lens.
				● Online module						
Li et al. (2019) USA	A mixed-method study	To evaluate the placement of EOLC education and students' experience through ELNEC training	37 senior students	● Online course				Curriculum integrating an online series of ELNEC modules. The curriculum includes ethical considerations, cultural considerations, communication, grief, and final hours.	Uniquely designed questionnaire items: 6 questions regarding sociodemographic information and 6 questions regarding experience in EOLC education	Students’ attitudes toward death and dying can be improved through the integration of EOLC education in the curriculum. The students had received the ELNEC core modules throughout the four years of education, and every level was suitable for the ELNEC curriculum placement from junior to senior level, with the exception of the freshman level.
Mal (2015) USA	Two-arm pre-post test study	To compares two methodologies used in teaching EOL	24 senior students	●			Case study	Comparison between the group that received the lecture followed by the case study and the group that received the lecture followed by the simulation. Ethics and cultural influences included in lecture content.	A computerized question exam developed by the Health Education Systems, Inc (HESI) measuring content knowledge gain, critical thinking, and comfort levels with EOLC.	The results of evaluation tools supported the inference that the simulation group felt more challenged, confident, and better prepared to care for a dying patient after participating in the research than the case study group. The results of this study showed a marginal increase in the mean scores of the simulation group as compared to the case study group. The students involved in the experiential learning experience showed an enhanced awareness of the patient’s and family’s needs and demonstrated a higher level of compassion than the case study group.
				●	● Same scenario as in the case study					
Mason et al. (2020) USA	Quasi-experimental study	To examine the effect of an interactive, multimodality palliative care class	18 students No information on grades	●	●		Games Small group discussions Computerized learning modules Readings Reflection	Ethics and culture are included in the educational content.	PCQN; FATCOD	PCQN results showed an improvement in post-test scores. Student-completed reflection papers on their learning experiences showed an improvement in students' attitudes, knowledge, and comfort with palliative and EOLC. The FATCOD results showed a significant improvement in comfort scores at post-test. This demonstrates the value of using a multimodality approach based on Kolb's Experiential Learning Theory.
Orr et al. (2021) USA	Quasi-experimental study	To assess the feasibility, effectiveness, and satisfaction of a workshop covering the principles of EOLC	19 senior students, 23 RN, and one NP	●				ELNEC Core “Train-the-Trainer” courses.	ELNEC–KAT	ELNEC-KAT scores were significantly higher after the workshop compared to pre-workshop scores, indicating that students gained knowledge from the workshop.
Rauch et al. (2023) USA	Quasi-experimental study & mixed-method study	To examine students' self-efficacy for conducting conversations with patients with serious illnesses and to describe students' perceptions	31 senior students (licensed and unlicensed)				In-class activity In-community practice	Educational interventions engaging in conversations with critically ill patients. Activity involved use of statement cards that provided scenarios of possible patient statements along with communication prompts to help respond to and initiate serious illness conversations with older adult clients in the community settings.	Uniquely designed questionnaire items Questions about change in confidence	Students reported an improved perception of their ability to conduct serious illness conversations and emphasized the need to practice these conversations in clinical practice.
Robinson et al. (2017) USA	Two-arm pre-post test study	To examine knowledge and attitudes in a program toward EOL care following completion of a course modeled on the ELNEC	74 senior students	●				Comparison between those who received the palliative care course modelled on the ELNEC and those who did not. EOLC elective course based on ELNEC with ethical issues in the content.	PCQN; FATCOD	When comparing the PCQN scores of the attending and nonattending groups, the attending group scored significantly higher and gained more knowledge. Scores on the FATCOD scale, which measures attitudes toward EOLC, were also significantly higher in the attending group, and the Pearson correlation coefficient between the FATCOD scale and PCQN scores showed a weak but significant positive correlation (*r* = 0.257, *P* = 0.027) between students' attitudes and knowledge about EOLC.
			
Sarabia-Cobo et al. (2016) Spain	Quasi-experimental study & mixed-method study	To evaluate a learning intervention in palliative care using a LFS	68 sophomore students	●	●			The content of ethical and legal issues was taught in pre-simulation lectures.	Uniquely designed questionnaire items	The use of two scenarios, one with actors and one with a low-fidelity human simulation, helped students connect theory with practice. It also gave them the opportunity to train how to approach family management and secure a safe environment for the patient.
Smothers et al. (2019) USA	Quantitative longitudinal study	To develop an understanding of prelicensure nursing students’ attitudes toward care for dying patients	40 sophomore to senior students	●				ELNEC core curriculum	FATCOD	Of the 30 questions on the FATCOD, the mean differences were positive for eight questions, including "I would be uncomfortab le talking about impending death with the dying person,” and “I would not want to care for a dying person”, indicating that seniors' responses tended toward "strongly disagree." For six questions, such as “Giving care to the dying person is a worthwhile experience,” and “Caring for the patient’s family should continue throughout the period of grief and bereavement,” the difference in mean scores was negative, indicating that the seniors' responses tended toward "strongly agree." The more advanced students showed more positive feelings about caring for the terminally ill.
Valen et al. (2022) Norway	Quasi-experimental study	To examine students’ attainment of learning outcomes in palliative care through simulation and hospital placement	55 sophomore students		●	●		3 h of simulation based on the International Nursing Association for Clinical Simulation and Learning Standards of Best Practice and hospital practice.	Uniquely designed questionnaire items	Wilcoxon signed-rank test showed positive differences in the pretest and poststimulation test evaluation for all questions, indicating that overall, the simulation affected knowledge, skills, and competence.
van der Wath et al. (2015) South Africa	Qualitative descriptive study	To explore and describe students’ experiences of EOLC through experiential learning within a constructivist educational model	64 sophomore students				Experiential learning	Experiential learning (presenting their experiences on a theme related to death and dying)	Analyze qualitative descriptions	Participants experienced heightened emotional awareness as they reflected on their own encounters with death and dying. Participants were challenged to clarify their values and realize their obligation to respect others’ values. They perceived themselves as more competent to care for dying patients and bereaved families with compassion and empathy.
Wu et al. (2023) China	Two-arm pre-post test study & mixed-method study	To determine the effect of death education course using constructivist learning theory on students' attitudes and coping abilities toward death	191 freshmen	●			Group discussion Role-play Sharing meeting	Ethics of death is integrated into the course content, and perspectives that require students to write reflections include "Have your views on ethics/death changed?", "Why? What do you think?" as perspectives from which students are asked to write reflections.	DAP-R; CDS; PCQN	Students who participated in the death education course utilizing constructivist learning theory had significantly greater levels of death acceptance, coping ability, and psycho-spiritual social support than those in the control group.
				●						

CAT, Communication Assessment Tool; CDS, Coping with Death Scale; DAP-R, Death Attitudes Profile-Revised; DAS, Death Anxiety Scale; DNR, do not resuscitate; EHC-PS, Health Professionals Communication Skills Scale; ELNEC, End-of-Life Nursing Education Consortium; ELNEC-KAT, ELNEC-Knowledge Assessment Test; EOL, end of life; EOLC, end of life care; FATCOD, Frommelt Attitude Toward Care of the Dying Scale; HFS, high-fidelity simulation; LFS, low-fidelity simulation; PCQN, Palliative Care Quiz for Nursing; RCT,randomized controlled trial; RN-BSN, Registered Nurse-Bachelor of Science in Nursing; SE-12, Self-Efficacy questionnaire; SP, standarlized patient; SPS, standarlized patient simulation.

Almost all studies included lectures as a teaching method, including traditional lectures, online lectures, and video learning. Furthermore, some studies applied group discussions, case studies, role-play, or attempted to create a simulated funeral home. Some also introduced the ELNEC educational program. In addition to lectures, some studies included hospital training and simulations. The simulations included high- and low-fidelity simulations and simulated patients. Of these, 17 studies used lectures to teach ethics in EOLC, and four also used scenarios and case studies. Twelve studies used scenarios for simulation education—seven studies combined lectures with simulations.

### 4.2 Educational methods and their effectiveness

#### 4.2.1 Lecture

Of 17 studies, seven incorporated teaching methods that included an ELNEC module; the ELNEC core module included the content of “Ethics in Palliative Care” in Module 4. Four studies integrated this content (Glover et al., 2017; Li et al., 2019; Smothers et al., 2019; and Orr et al., 2021). Three did not incorporate module 4 but taught their ethics content (Conner et al., 2014; Robinson and Epps, 2017; Alwawi and İnkaya, 2023). Conner et al. (2014) reported that an online "Death and Dying" course improved nursing students' attitudes toward EOLC, and the students who took the EOLC course improved their knowledge and attitudes toward EOLC (Robinson and Epps, 2017; Alwawi and İnkaya, 2023). Of the remaining 10 studies, the lecture topics included ethical decision-making (Conner et al., 2014; Loerzel and Conner, 2016; Goode et al., 2019), ethical issues (Jo and An, 2015; Bassah et al., 2018), ethics and culture (Mason et al., 2020), ethical and legal issues (Sarabia-Cobo et al., 2016), ethics and cultural influences (Mal, 2015), ethics of death (Wu et al., 2023), and ethical and practical roles of nurses (Rauch et al., 2023). The students improved their knowledge about EOLC (Orr et al., 2021; Robinson and Epps, 2017), positive attitudes toward EOLC (Robinson and Epps, 2017; Smothers et al., 2019), confidence in EOLC (Loerzel and Conner, 2016), and attitudes toward death (Li et al., 2019).

#### 4.2.2 Simulation

Ten of the 12 articles presented clear ethical issues in simulation education (Grossman, 2013; Mal, 2015; Hjelmfors et al., 2016; Donnelly et al., 2017; Goode et al., 2019; Lewis-Pierre et al., 2019; Jablonski et al., 2020; Escribano et al., 2021; Valen et al., 2022; and (Alwawi and İnkaya, 2023) (Table 3). Three studies simulated situations involving breaking bad news (Grossman, 2013; Escribano et al., 2021; Alwawi and Inkaya, 2022). Goode et al. (2019) included content on ethical decision-making, although not described in detail. Escribano et al. (2021) had content on family conflict decision-making, and Mal (2015) included content on decision support in situations where the family refused to inform the individual. Issues related to do not resuscitate (DNR) and the transition to comfort care were addressed in studies by Goode et al. (2019) and Jablonski et al. (2020). Valen et al. (2022) considered the patient's autonomy and integrity in the situation of recommending food to a patient who was terminally ill and not interested in eating and addressed content related to ensuring patient and family integrity and dignity in the situation of being diagnosed as terminal. Donnelly et al. (2017) addressed surrogate decision-making for patients who lacked capacity. In addition, one study included an ethical perspective in the debriefing of the simulation (Hjelmfors et al., 2016). As a result of these simulation-based educational interventions, there were improvements in knowledge (Grossman, 2013; Valen et al., 2022) and attitudes (Lewis-Pierre et al., 2019; Valen et al., 2022), improvements in self-efficacy and communication skills (Escribano et al., 2021), comfort in caring for those who are dying of a sudden illness (Grossman, 2013), feelings of challenge and confidence in care (Mal, 2015), and opportunities to reflect on one's attitudes and discuss the factors that impact them (Hjelmfors et al., 2016). There was no difference in knowledge between students who participated in the ethics consultation simulation and those who watched a video recording of the simulation (Donnelly et al., 2017). There were also no differences in knowledge between the standardized patient simulation (SPS) and a low-fidelity simulation, but the SPS group improved significantly in skills ((Alwawi and İnkaya, 2023); Jablonski et al., 2020; Mal, 2015)

**Table 3:** Ethical issues focused on EOLC simulation education.

Authors (year) Country	Ethical issues in focus	Case Details
Alwawi et al. (2023) Palestine	Giving bad news to the patient's family.	No detailed description.
Donnelly et al. (2017) USA	Patient’s lack of decision making capacity and the need for an appropriate decision maker. Nurse’s need to focus on patient as primary commitment and not to lose that focus on the face of potentially competing interests. The ethical obligation of the nurse to protect the patient from questionable care.	An elderly man who lacked decisional capacity and had a complicated medical course with respiratory failure secondary to pneumonia was admitted to the hospital from a long-term care facility. The surrogate decision maker was the patient’s adult child, although the patient’s sibling also wished to participate in the decision-making process. Concerns had been raised about the aggressive nature of the care given to the patient. The need to clarify goals of treatment was the reason that the ethics consultation was convened.
Escribano et al. (2021) Spain	Conflict in family decision making. Giving bad news.	No detailed description.
Goode et al. (2019) UK	DNR Transition to comfort care	No detailed description.
Grossman (2013) USA	Giving bad news	Three simulations, which included a 54-year-old woman with lymphoma who had become septic and was admitted to the medical intensive care unit, a 33-year-old man with cystic fibrosis who had contracted pneumonia after the flu and was experiencing leaky capillary syndrome, and a 34-year-old woman with severe heart failure secondary to complications of treatment from rheumatoid arthritis who experienced cardiac arrest almost daily and was adding a do-not-resuscitate status to stop further resuscitation efforts.
Hjelmfors et al. (2016) Sweden	Debriefing focuses on ethics, but does not list detailed ethical issues.	A high-fidelity mannequin played the role of the patient, a 40-year-old woman named Karin who was dying of cancer. The students met Karin in her home. She was lying in bed wearing a long T-shirt, underwear, trousers, and a scarf around her head. She had a stoma and a subcutaneous port-a-cath. She had terminal cancer and was expected to die soon.
Jablonski et al. (2020) USA	DNR Transition to comfort care	The scenario features a 77-year-old man who was introduced to the students in a previous simulation when he was admitted for an exacerbation of chronic lung disease. During his hospitalization, a chest x-ray revealed a small lung lesion, but he delayed follow-up to care for his ill wife. The patient subsequently experienced weight loss and increasing cough with hemoptysis and was diagnosed with metastatic lung cancer. He refused surgical interventions after diagnosis but underwent radiation therapy. He declined further treatment after his wife died. He was admitted to the medical unit for the management of unrelieved symptoms.
Lewis-Pierre et al. (2019) USA	No detailed ethical issues are listed.	Two-part hybrid simulations of a patient diagnosed with end-stage Non-Hodgkin's Lymphoma: full-code-status and DNR
Mal (2015) USA	Decision-making support. Family members who refuse to inform the individual.	**Scenario 1**. Jeremy S., 26-year-old male, case of stomach cancer. **Scenario 2**. Mr. McKinney, a 72-year-old male, malignant tumor with metastasis in lymph nodes or multiple organs. **Scenario 3**: Mei Lin C., a 30-year-old Asian female. Loss of a child due to uterine rupture. **Scenario 4**: Carly P., 17-year-old female. Car accident causing damage to the abdominal aorta and decision to donate organs.
Valen et al. (2022) Norway	Case1: Patient autonomy and integrity in the situation of recommending food to a patient who realizes he is dying and shows no interest in eating. Case2: Ensuring the integrity and dignity of patients and their families in situations diagnosed as terminal.	**Patient**: Jesper Jensen, 69 years old. Metastatic lung cancer. Hospitalized with poor general condition, pneumonia, and pain. Treated with antibiotics and analgesics. No longer interested in food. Informed by the doctor of short life expectancy. **Case 1: Relational skills.** The students simulate that the nurse is taking away the antibiotic infusion, and offer the patient some food. Jensen is tired. He has realized that he is going to die soon, and is no longer interested in eating. Jensen's wife has a different view of the situation and requests tube feeding for her husband. A teacher acted as the standardized patient. **Case 2: Clinical assessment.** This case focused on clinical assessment when Jensen is diagnosed as terminal. The wife is present. A High Fidelity Simulator is used.

#### 4.2.3 Role plays, discussions, case-studies, and other teaching methods

There were role plays in four papers, respectively (Bassah et al., 2018; Goode et al., 2019; Jo and An, 2015; and Wu et al., 2023), group discussions as reported in five papers (Bassah et al., 2018; Conner et al., 2014; Jo and An, 2015; Mason et al., 2020; and Wu et al., 2023), and case studies in four papers (Bailey and Hewison, 2014; Bassah et al., 2018; Grossman, 2013; and Mal, 2015). Other interventions included video/YouTube viewing (Bassah et al., 2018; Conner et al., 2014) and reflection on the educational experience (Conner et al., 2014; Mason et al., 2020). Specific educational content included interventions in which students watched simulation videos of other students' ethics consultation (Donnelly et al., 2017), interventions in which they built a model funeral home (Jo and An, 2015), and content in which they were asked to describe their own experiences with death and dying (van der Wath and du Toit, 2015). Some of these were implemented by combining several teaching methods, and not all strictly demonstrate the effectiveness of a single teaching method alone. However, episode-based case studies reportedly significantly improved nursing students' attitudes toward EOLC (Bailey and Hewison, 2014). As a result of experiential learning about death and death-related experiences, nursing students increased their emotional awareness while reflecting on themselves in the face of death and dying (van der Wath and du Toit, 2015). The intervention, which created a model funeral home and incorporated discussion, improved students' positive attitudes toward death and communication skills (Jo and An, 2015). Mason et al. (2020) demonstrated the value of a multimodal approach.

#### 4.2.4 Clinical practice

One study involved clinical practice. In the study by Valen et al. (2022), the students improved their knowledge, skills, and attitudes after a simulation intervention during an eight-week hospital practice. But it is unclear whether these have improved as integrated clinical skills.

### 4.3 Evaluation instruments

In all studies that assessed knowledge of the Palliative Care Quiz for Nursing (PCQN), a 20-item test designed to assess nurses' knowledge of palliative care (Ross and McGuinness, 1996), and the ELNEC-Knowledge Assessment Test (ELNEC-KAT) (Lange et al., 2009) that is a 50-item tool based on the ELNEC curriculum, students who received some type of educational intervention gained more knowledge than before the intervention (Robinson et al. 2017; Mason et al. 2020, Alwawi and İnkaya, 2023, Wu et al. 2023, Grossman 2013, Glover et al. 2017, and Orr et al. 2021). Wu et al. (2023) reported that the group that received role-playing and discussion increased their knowledge of the psychosocial aspects of the PCQN more than the group that received traditional education. In a study by Donnelly et al. (2017). that evaluated items from the Guide to the Code of Nursing Ethics with Interpretations, written by Marcia Fowler, Ph.D., they reported no difference in knowledge between the simulation group and the group that watched a video of the simulation.

The Frommelt Attitude Toward Care of Dying (FATCOD) and FATCOD-B scale were used as indicators to measure student attitudes. Studies evaluating the educational effectiveness of these scales have reported more positive attitudes toward care of the dying with educational interventions (Bailey et al. 2014; Conner et al. 2014; Robinson et al. 2017; Lewis-Pierre et al. 2019; Smothers et al. 2019, and Mason et al. 2020). Among these, Lewis-Pierre et al. (2019) found that the addition of the simulation, as well as the online module, made the experience more holistic and meaningful for students, who were able to develop a personal understanding of death and dying and begin to care for these individuals and their families through a constructive lens.

No common methods for measuring student skills were found. Escribano et al. (2021) measured communication skills using the Health Professionals Communication Skills Scale (EHC-PS) and reported that, although no significant differences were observed after eight 2.5-hour simulations with simulated patients, communication skills had improved. Alwawi et al. (2023) compared a low-fidelity simulator simulation group with a simulated patient group and reported that the simulated patient group, using uniquely designed questionnaire items, had significantly better skills.

In addition, there have been studies that have used qualitative analysis to explore the impact on students' learning. Hjelmfors et al. (2016) report that the EOLC simulation provided nursing students with training in communication skills in difficult situations and an opportunity to reflect on their attitudes and feelings and discuss what factors influence such conversations. Van der Wath et al. (2015) report that experiential learning reflections challenged students to articulate their values but also made them aware of their obligation to respect the values of others.

## 5 Discussion

### 5.1 EOLC ethics education in baccalaureate of nursing education

Undergraduate students often have limited experience of end-of-life care; there are very few opportunities for students in undergraduate education programs to practice the application of knowledge and skills in EOLC practice settings (show how, do), as students are required by law to have a fixed number of minimum hours of practice (Li et al., 2019). Many universities worldwide have tried EOLC education to increase understanding and develop positive attitudes toward death (Lippe and Carter, 2015). Our findings show that many studies included some ethical content of EOLC in undergraduate nursing education in different countries. However, no studies focused on ethics education in EOLC to help students acquire ethical competence.

Death-related content permeates other undergraduate nursing courses, such as basic nursing and nursing humanities (Andersson et al., 2022). Two approaches to teaching ethics in EOLC can be considered: 1) incorporating EOL topics when teaching nursing ethics, and 2) integrating ethical content when teaching EOLC. Incorporating EOL topics would allow education by faculty specializing in nursing ethics. In contrast, integrating ethical content would enable students to learn about ethical aspects while studying the physical and psychosocial conditions of EOL patients. The education identified in the articles reviewed was predominantly of the latter type, with a strong emphasis on pain and symptom management throughout the curriculum. In medical ethics education, it has been demonstrated that learning ethical competencies is essential for paying attention to ethical issues and dealing with them effectively (Andersson et al., 2022).

According to Miller's pyramid of hierarchical learning, the cognitive levels and application of knowledge (know and know-how, respectively) support the practical application of that knowledge (show how) and the ability to act (do) to apply what is learned in practice (Miller, 1990). Addressing ethical issues in EOLC is complex (Alanazi et al., 2024). Collecting information related to ethical issues, interpreting the needs and actions of stakeholders (e.g., patients, families, other healthcare professionals), and identifying and justifying ethical issues are components of nursing ethics education (Greco et al., 2019). Students must apply this knowledge in practice to apply ethical judgments in diverse nursing roles and clinical settings. The results of this study showed that lecture-based educational methods were generally reported as effective in acquiring knowledge (know), but incorporating role-playing and discussion was more effective in gaining knowledge about psychosocial aspects (Wu et al., 2023). Applying ethical principles and theories to ethical issues seems to involve applying knowledge (know-how). Conversely, case studies, discussions, and clinical conferences based on case examples help make ethical judgments/decisions. Even based on Kolb's experiential learning theory (Kolb, 2014), which focuses on the importance of the learner moving through four stages, a class structure that incorporates a multimodal approach, including games, simulations, and small group discussions, in addition to lectures, is effective in improving students’ attitudes and knowledge of EOLC. The results of this study suggest that communication skills in limited situations, such as "giving bad news," can be acquired through role-playing and simulation. As for ethical practice, it is not about learning specific nursing skills, live communication, and attitudinal training using role-playing among students and simulated patients may be more effective than simulations using a mannequin (Alwawi and İnkaya, 2023; Jablonski et al., 2020; Mal, 2015). As palliative care involves complex decision-making processes such as withdrawing or withholding life-sustaining treatment and delivering bad news, it is important to integrate communication skills into nursing curricula and link real-life case studies with learning opportunities (Ramjan et al., 2010).

### 5.2 EOLC ethical education: learning goals and student support

Ethics are required in professional nursing practice, not just in EOLC. It is specifically stated in The Essentials: Core Competencies for Professional Nursing Education (American Association of Colleges of Nursing, 2021) as an element of professionalism, which is a core competency.

End-of-life care education (EOLC) involves many ethical dilemmas related to death, so it is necessary to carefully consider students' attitudes toward death and their perceptions of life and death to understand and educate them (Zhu et al., 2022). Further education on loss, grief, bereavement, and the final hours is required. Nursing educators are responsible for providing the best educational methods to ensure that students can perform effectively, safely, and receive the necessary care (Alwawi and İnkaya, 2023). Because EOLC is a complex situation in which students lack experience, learning needs to be reinforced through lectures, simulations, clinical practice and other experiential learning activities. Furthermore, educational opportunities for nursing educators are also important to implement such education.

International training programs for EOLC (such as the ELNEC project) have been implemented in several countries (Ferrell et al., 2015). Courses are not limited to face-to-face sessions but are being made more widely available through online education. However, little is known about which features of educational interventions are most effective in medical ethics education and their long-term outcomes (Moreira et al. 2021). There is no consensus on the level of competence undergraduate students should aim to achieve in ethical education related to EOLC. We believe that further research can focus on evaluating the development of educational programs to improve nursing students' attitudes and coping skills for death and provide new insights into international death education. The educational approach will increase the preparedness of undergraduate nursing students for the complex EOLC involving the physical, social, psychological, and spiritual needs of patients.

## 6 Strengths and limitations

This scoping review had several strengths and limitations. One strength was that the search strategy was developed following discussions with experienced researchers, and a thorough literature search was conducted.

However, due to language limitations, relevant studies may have been excluded. The review did not utilize literature search databases in fields such as sociology or philosophy, which resulted in a limited scope. Furthermore, as the definitions of terms related to end-of-life care education programs are not standardized, the educational and clinical implications should be interpreted with caution.

Future research should consider the terminology used in end-of-life care and conduct additional studies that consider culturally diverse aspects.

## 7 Conclusion

This review aimed to examine how EOLC ethics education is delivered in undergraduate nursing education and describe the outcomes of ethics education. Nurses face ethical dilemmas and conflicts in EOLC, and addressing these issues in undergraduate education is important. This review found that (a) EOLC ethics education in undergraduate nursing education includes ethical content as part of EOLC nursing education; however, no study has specifically examined it; (b) undergraduate EOLC education is effective in preparing students for complex and individualized EOLC nursing practice; and (c) it is important to understand that knowledge of EOLC ethics can be acquired through lectures, but using this knowledge to make ethical decisions and practice ethical behavior requires multimodal education that incorporates role-playing scenarios, discussions, and simulations. In the future, it would be desirable to establish curricula and educational programs that allow for more systematic ethics education in EOLC.

## Funding

This study was supported by the project of Yamaguchi University Priority Universities for cooperation.

Supplementary data

Supplementary data to this article can be obtained from the corresponding author upon request.

## CRediT authorship contribution statement

**Saeko Kutsunugi:** Writing – original draft, Visualization, Resources, Methodology, Investigation, Formal analysis, Data curation, Conceptualization. **Satoko Ono:** Writing – original draft, Visualization, Resources, Methodology, Investigation, Formal analysis, Data curation, Conceptualization. **Misae Ito:** Writing – original draft, Visualization, Resources, Methodology, Investigation, Formal analysis, Data curation, Conceptualization. **Kaho Suda:** Visualization, Methodology, Investigation, Data curation, Conceptualization. **Siu Ling Chan:** Data curation, Conceptualization. **John Tai Chun Fung:** Data curation, Conceptualization. **Claudia Kam Yuk Lai:** Writing – review & editing, Data curation, Conceptualization. **Kyoko Murakami:** Supervision, Funding acquisition, Conceptualization.

## Declaration of competing interest

The authors declare the following financial interests/personal relationships which may be considered as potential competing interests: Kyoko Murakami reports financial support was provided by Yamaguchi University Priority Universities project for cooperation. If there are other authors, they declare that they have no known competing financial interests or personal relationships that could have appeared to influence the work reported in this paper.

## Data Availability

All data in this study are available in the published literature and are included in this article.
